# Spatiotemporal analyses of the epidemiological characteristics of diabetes mellitus

**DOI:** 10.4178/epih.e2021102

**Published:** 2021-12-16

**Authors:** Sang Youl Rhee

**Affiliations:** Department of Endocrinology and Metabolism, Kyung Hee University School of Medicine, Seoul, Korea

**Keywords:** Spatio-temporal analysis, Epidemiologic study characteristics, Diabetes mellitus, Chronic disease

## Abstract

Research based on spatiotemporal analysis has been conducted to identify various factors that can affect an individual’s or community’s degree of health and disease. These spatiotemporal studies can effectively illustrate patterns in disease frequency, features, and temporal flow in different parts of a country. Furthermore, identifying these regional characteristics can aid in the development of disease prevention or intervention strategies.

## DISEASE ANALYSIS ON A SPATIAL AND TEMPORAL SCALE

Research using spatiotemporal analysis techniques has been conducted to identify various factors that can affect an individual’s or community’s degree of health and disease [1]. Several studies have demonstrated temporal and spatial connections of severe or chronic diseases utilizing analytical approaches such as spatial autocorrelation, ordinary least squares, or geographically weighted regression. These spatiotemporal studies can effectively illustrate patterns in disease frequency, features, and temporal flow in different parts of a country. Furthermore, identifying regional characteristics can aid in the development of disease prevention or intervention strategies [2].

### Spatiotemporal trends in diabetes-related outcomes

The study by Nurjannah et al. [3] published in this issue of Epidemiology and Health investigated the spatiotemporal dynamics of diabetes-related mortality according to regional boundaries in Michigan, United States. This study used school districts as the geographic unit of analysis, and the study was conducted using death records from 2007 to 2014. The study demonstrated the existence of a regional cluster with significantly higher diabetes-related mortality in Michigan.

This study is meaningful in that it suggested spatial and temporal disease hotspots by utilizing school districts as a regional factor. Using these hotspots, we can reach a deeper spatiotemporal understanding of the disease burden associated with diabetes mellitus (DM) in our communities. The authors argued that the findings of their study could help prioritize the establishment of school-based DM prevention programs in the community.

The results of this study suggest that the spatial distribution of DM and related outcomes is not random; instead, it is regionally specific and can show unique interactions depending on geographic proximity. On a similar note, Grubesic et al., [4] suggested that place can be a basis for health and unhealth, and argued that rural and low-income areas and physical environmental factors affect health levels. Dijkstra et al. [5] in a study of drug use in people with type 2 DM in the Netherlands, found that living in an aging community and belonging to low-income groups may influence the incidence of DM. Using spatial analysis, Bocquier et al. [6] confirmed that the degree of aging and low economic levels in southeastern France were related to DM.

## RELATED RESEARCH TRENDS IN KOREA

In Korea, studies on geographic variables of DM have centered on administrative districts such as cities, counties, and districts. Kim et al. [7] confirmed that the prevalence of DM varied by region using stepwise regression analysis and decision trees. Other researchers also found that the prevalence of DM was significantly higher in rural areas and regions with a high proportion of the elderly population in Korea [8]. These results are similar to those of other studies abroad that analyzed the relationships of the degree of aging and socioeconomic level with DM at the regional level. However, not many studies in Korea have considered geographic factors other than administrative districts or studied the characteristics of DM-related outcomes. There are also not many longitudinal studies that have analyzed trends over time.

## LIMITATIONS OF SPATIOTEMPORAL ANALYSIS

Despite its many advantages, spatiotemporal research has inherent limitations. First, it is difficult to fully consider the diversity within regional units because the research is conducted under the assumption that the characteristics of each regional unit are uniform. Second, since only some of the variables representing regional characteristics are included in the study, it may be difficult to fully explain the study results. Third, this study design has the potential to cause ecological errors when interpreting the results. Therefore, interpretation of the findings in terms of individual characteristics is almost impossible. It is necessary to fully consider these limitations when interpreting study results.

## DISCUSSION

According to the Korean Diabetes Association’s “Diabetes Fact Sheet of Korea,” 13.8% of adults in Korea over the age of 30 have DM [9]. Various chronic complications occur in people with DM. These chronic complications of diabetes are known to significantly reduce individuals’ quality of life. Furthermore, they may result in exorbitant medical expenses, thus jeopardizing the healthcare system’s stability. A disease burden analysis utilizing disability-adjusted life years found that DM was the disease with the highest burden in Korea [10]. In Korea, around 25% of people over 30 years of age have impaired fasting glucose, and the public health crisis caused by DM is predicted to rise in the future [9]. In the current situation where active measures for the effective management and prevention of diabetes need to be sought, spatiotemporal studies can make a valuable contribution to the decision-making process.

### Ethics statement

This paper is an editorial, so it did not need ethical approval.
